# Fermented ginseng powder enriched with rare ginsenosides ameliorates high-fat diet-induced obesity by modulating adipogenesis and inflammation

**DOI:** 10.29219/fnr.v69.12230

**Published:** 2025-05-08

**Authors:** Xueyue Tai, Jiating Li, Jianwei Song, Bao Zhong, Fenglin Li

**Affiliations:** 1 College of Food Science and Nutritional Engineering, Jilin Agriculture Science and Technology University, Jilin, China; 2 College of Food Science and Engineering, Changchun University, Changchun, China;; 3 School of Public Health, Jilin Medical University, Jilin, China;; 4 Brewing Technology Innovation Center of Jilin province, Jilin Agriculture Science and Technology University, Jilin, China

**Keywords:** fermented ginseng, obesity, high-fat diet, lipid-lowering, modulating effect

## Abstract

Administration of high-dose fermented ginseng powder (2.385 mg/g) resulted in a reduction in body weight and an improvement in blood biochemical parameters in high-fat diet (HFD)-fed mice. Significant reductions in lipid droplet size were observed in both liver and epididymal adipose tissues. Western blot analysis showed increased protein levels of PPAR-α, PPAR-γ, and PGC-1 in the HFD + low-dose lyophilized fermented ginseng powder (HDL), HFD + medium-dose lyophilized fermented ginseng powder (HDM), and HFD + high-dose lyophilized fermented ginseng powder (HDH) groups compared to the HD group. Furthermore, the phosphorylation of AMPK (P-AMPK) and ACC (P-ACC) was significantly elevated. Conversely, western blot analysis demonstrated a decrease in the expression of inflammatory cytokines IL-1, IL-6, and TNF-α in the CG, HDL, HDM, and HDH groups compared to the HD group. Gene expression analysis revealed a downregulation of lipid anabolism-related genes, including *SREBP-1c* and *FAS*, along with an upregulation of *PPAR-γ* and *ACOX-1* mRNA levels. Additionally, the expression of inflammation-related genes such as *IL-1*, *IL-6*, and *TNF-α* was reduced. High-dose freeze-dried fermented ginseng powder (2.385 mg/g) significantly influenced lipid metabolism and inflammatory responses, highlighting its potential as a therapeutic agent for the management of dyslipidemia.

## Popular scientific summary

- A fermented ginseng powder, enriched with rare ginsenosides, affects obesity and inflammation in mice fed a high-fat diet.
- Mice treated with fermented ginseng showed reduced body weight and improved blood fat and glucose profiles.
- Liver and abdominal fat tissue was significantly reduced.
- Important proteins that help burn fat and improve metabolism (e.g., PPAR-α, PPAR-γ, PGC-1, P-AMPK, and P-ACC) were increased.
- Inflammatory markers (IL-1, IL-6, TNF-α) in both protein and gene expression levels were decreased, indicating an anti-inflammatory effect.
- Genes involved in fat production (e.g., SREBP-1c and FAS) were downregulated, while those promoting fat breakdown (e.g., PPAR-γ and ACOX-1) were upregulated.

Obesity is a chronic metabolic disease caused by the excessive accumulation of body fat in an organism, resulting in excessive weight gain and abnormal fat distribution, and can lead to several chronic diseases, such as diabetes mellitus and cardiovascular disease (1, 2). High-fat diet (HFD)-induced obesity is one of the most commonly used animal models to study the pathophysiological mechanisms underlying obesity and its related complications (3). This model induces adipogenesis, the process of fat cell formation and triggers systemic inflammation, both of which are key contributors to the development and progression of obesity (4). HFD-induced obesity is widely used as an experimental model to study the pathophysiology of obesity and its associated complications (5). Adipogenesis, the process of fat cell formation, and inflammation are central to the development of obesity and its related disorders (6). Inflammation, particularly chronic low-grade inflammation, is considered a critical factor in the progression of obesity, as it exacerbates insulin resistance and impairs lipid metabolism (7). Therefore, therapeutic strategies targeting both adipogenesis and inflammation are essential in combating obesity and related diseases.

Ginseng (*Panax ginseng C.A. Meyer*), a traditional herbal medicine, has long been used in East Asia for its wide range of therapeutic effects, including its anti-inflammatory, anti-obesity and metabolic regulatory properties (8). The bioactive compounds in ginseng, primarily ginsenosides, have been shown to exert beneficial effects on adipocyte differentiation, lipid metabolism, and inflammation (9). However, the biological activities of ginsenosides are known to be influenced by their chemical structure, and recent studies have highlighted the increased bioavailability and enhanced biological activities of rare ginsenosides, such as Rh2, Rg3, and Rk1, which are produced through fermentation processes (10). Fermented ginseng has been shown to possess improved pharmacological properties compared to non-fermented ginseng, including greater efficacy in regulating metabolic functions and inflammation (11). Fermentation can increase the content of rare saponins in ginseng (12). Studies have shown that fermentation can alter the sugar chain structure of ginsenosides to promote the conversion of conventional saponins into rare saponins in ginseng; the fewer sugar groups, the stronger the physiological activity (13). *Lactobacillus* fermentation produces β-glucosidase, an enzyme that modifies glycosidic bonds within ginsenoside molecules. Through the hydrolysis of various β-glucosides by *Lactobacillus*, this process not only generates rare saponins with enhanced activity and medicinal properties, but also incorporates *Lactobacillus* metabolites into the fermented ginseng. Consequently, it enhances both the functionality and nutritional value of ginseng (14, 15). The impact of fermented ginseng on obesity and metabolic disorders has gained considerable attention in recent years. Studies have suggested that fermented ginseng can mitigate HFD-induced obesity by regulating adipogenesis and modulating inflammatory responses (16). These effects are mediated through key signaling pathways such as the peroxisome proliferator-activated receptors (PPARs), AMP-activated protein kinase (AMPK), and acetyl-CoA carboxylase (ACC), which play crucial roles in lipid metabolism, adipocyte differentiation, and inflammation (17). Despite the promising findings, the mechanisms through which fermented ginseng powder enriched with rare ginsenosides affect obesity remain inadequately explored.

In the present study, we aimed to investigate the potential of fermented ginseng powder enriched with rare ginsenosides in ameliorating HFD-induced obesity. Specifically, we focused on its effects on adipogenesis and inflammation, with an emphasis on the modulation of key molecular pathways involved in lipid metabolism and inflammatory responses. By elucidating the molecular mechanisms underlying its anti-obesity effects, this study contributes to the understanding of fermented ginseng as a potential therapeutic agent for the management of dyslipidemia and obesity-related disorders.

## Materials and methods

### Determination of the saponin content in fermented ginseng and preparation of lyophilized powder

The ginseng stems powder was suspended in a skimmed milk powder medium and autoclaved at 121°C for 15 min. After inoculation with *Lactobacillus bulgaricus*, fermentation was carried out with an inoculum size of 3%, at a temperature of 40°C, for 72 h, with a pH maintained at 5. At the end of the fermentation process, the broth was extracted three times with saturated n-butanol. The n-butanol layers were then combined, concentrated, and the saponin content was quantified using high-performance liquid chromatography (HPLC). Finally, the fermented broth was lyophilized to obtain the fermented ginseng lyophilized powder, which was subsequently diluted with distilled water and stored.

### Animal experimental procedures

Fifty male ICR mice (Yisi Laboratory Animal Technology Co., Ltd., Changchun, China; age: 2–3 weeks; initial body weight: 20 ± 1.0 g; housed at 22 ± 2°C) were acclimatized to the laboratory environment for 1 week prior to the experiment. An obesity model was established by feeding the mice a HFD containing 60% of calories derived from fat. Experimental treatment commenced simultaneously with dietary intervention. The animals were randomly assigned to five experimental groups (*n* = 10 per group): control diet group (CG), HFD group (HD), HFD + low-dose lyophilized fermented ginseng powder (1.395 mg/g) (HDL), HFD medium-dose lyophilized fermented ginseng powder (1.860 mg/g) (HDM), and HFD + high-dose lyophilized fermented ginseng powder (2.385 mg/g) (HDH). The CG and HD groups were administered 0.1 mL of distilled water. Food intake and body weight were monitored weekly. At the end of the 12-week treatment period, the animals were fasted for 12 h before euthanasia. Blood, liver, and adipose tissues were collected and stored at –80°C for subsequent analysis.

### Oral Glucose Tolerance Test (OGTT)

After 12 weeks of continuous gavage, mice were fasted for 12 h (with no food or water) and then orally administered 2 g/kg of glucose at night. Blood samples were collected via tail vein at 0, 30, 60, and 120 min post-administration. Glucose concentrations were measured at each time point using a glucometer. The area under the glucose curve (AUC) was calculated using the trapezoidal rule.

### Biological parameter analysis

The levels of total cholesterol (TC), triglycerides (TG), high-density lipoprotein cholesterol (HDL-C), and low-density lipoprotein cholesterol (LDL-C) were measured automatically using an ELISA kit (Built-in Institute of Biological Engineering, Nanjing, China). Tumor necrosis factor-alpha (TNF-α), interleukin-6 (IL-6), and interleukin-1 (IL-1) were quantified using an ELISA kit (Jingmei Biotechnology Co., Ltd., Jiangsu, China).

### Histopathological analysis

The liver and epididymal fat were collected by dissection, washed with saline, and immediately fixed in 4% formalin for 24 h. Conventional paraffin-embedded tissue sections were then prepared. The sections were deparaffinized using xylene and ethanol, followed by washing with deionized water for 5 min. Hematoxylin and eosin (H&E) staining was performed (Leagene Biotechnology Co., Ltd., Beijing, China), and images were acquired using a microscope to observe morphological changes in the liver and adipose tissue.

### Western blotting

Liver tissue (100 mg) was added to a mixed lysate at a ratio of 1:10 g/mL. The supernatant was immediately extracted by centrifugation at 10,000 r/min in a high-speed centrifuge at 4°C for 10 min. The total protein content was determined using a BCA protein quantification kit (Tiangen Biochemical Technology Co., Ltd., Beijing, China). Proteins were subjected to SDS-PAGE (Epizyme Biomedical Technology Co., Ltd., Shanghai, China), electro-transferred to a Polyvinylidene (PVDF) membrane (Merck Millipore, Darmstadt, Germany), blocked in 5% bovine serum albumin (BSA) (Solarbio Technology Co., Ltd., Beijing, China) for 1 h, and incubated with anti-PPAR-α, anti-PPAR-γ, anti-AMPK, anti-P-AMPK, anti-PGC-1,anti-ACC,anti-P-ACC (Immunoway, Texas, USA), anti-TNF-α, anti-IL-1, or anti-IL-6 (Solarbio Technology Co., Ltd., Beijing, China) primary antibodies overnight at 4°C. The membranes were then rinsed five times with Tris-Buffered Saline Tween-20 (TBST). Secondary antibody was added and incubated at room temperature in the dark for 1 h, rinsed five times with TBST. An Excellent Chemiluminescent Substrate (ECL) chemiluminescent agent (Proteintech, California, USA) was developed and exposed for imaging, and relative protein expression was calculated by grayscale analysis of protein bands using ImageJ software (National Institutes of Health, Bethesda, MD, USA) with β-actin as an internal reference.

### Real-time polymerase chain reaction analysis

Total RNA was extracted utilizing TRIzol, and the extracted RNA was counted using a NanoDrop (Thermo Fisher, MA, USA). The cDNA was synthesized using the FastKing cDNA First Strand Synthesis Kit, and qPCR was performed using SYBR green qPCR mixture (Tiangen Biochemical Technology Co. Beijing, China). The PCR primers for the *SREBP-1C* (Sense 5’AAGCAAATCACTGAAGGACCTGG-3’, Antisense5’- AAAGACAAGGGGCTACTCTGGGAG-3’), *FAS* (Sense5’ AGGGGTCGACCTGGTCCTCA-3’, Antisense5’-GCCA TGCCCAGAGGGTGGTT-3’), *ACOX-1* (Sense5’-TAT TCGGCTATGACTGGGCACA-3’, Antisense5’-GATGGAT ACTTTCTCGGCAGGA-3’), *PPAR-α* (Sense 5’ GGATG TCACACAATGCAATTCGCT-3’, Antisense 5’TCACA GAACGGCTTCCTCAGGTT-3’), *TNF-α* (Sense 5’-ATGG CCCAGACCCTCACA-3’, Antisense5’-TTGCTACGACG TGGGCTACA-3’), *IL-6* (Sense5’-GCTTAATTACACA TGTTCTCTGGGAAA-3’, Antisense5’-CAAGTGCATCA TCGTTGTTCATAC-3’), *IL-1* (Sense5’-GACCTTCCAGG ATGAGGACA-3’,Antisense5’-AGCTCATATGG GTCCGACAG-3’), and *β-actin* (Sense5’-AGCCTTCCTT CTTGGGTATGG-3’,Antisense5’-CACTTGCGGTGCAC GATGGAG-3’) were used (Comatebio Biotechnology Co., Ltd., Changchun, China). PCR thermocycling conditions were denaturated at 95°C, 15 s; annealing at 60°C, 20 s; and extension at 72°C, 35 s, with a final extension at 72°C, 35 s for a total of 45 cycles.

### Statistical analysis

All data are presented as means ± standard deviation. Statistical analyses were conducted using SPSS software (v.23.0; SPSS Inc., Chicago, IL, USA), and differences among groups were assessed using one-way analysis of variance (ANOVA). Significant variations were further analyzed with Duncan’s Multiple Range test (*P* < 0.05). Statistical significance between groups was denoted by different letters (a, b, c, d, and e), where ‘a’ indicates the highest value and ‘d’ the lowest.

## Results

### Chromatogram of fermented ginseng

**Fig. 1:**
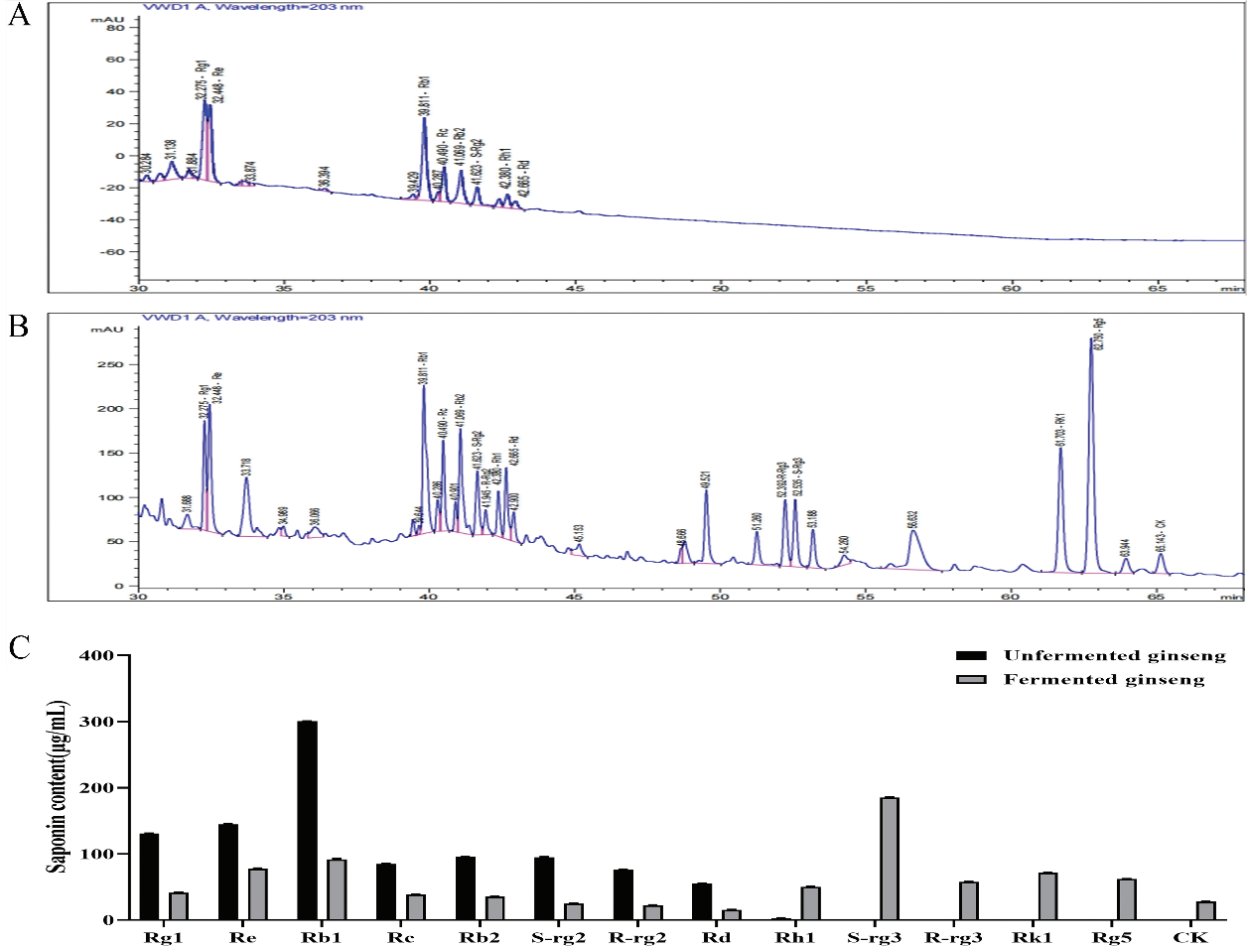
HPLC chromatograms depicting changes in saponin profiles of ginseng before and after fermentation by *Lactobacillus bulgaricus*. (A) Liquid chromatogram of ginseng saponin content before fermentation, (B) Liquid chromatogram of the saponin content of ginseng after fermentation, (C) Comparison of ginseng saponin content before and after fermentation.

As illustrated in Fig. 1, the fermentation of ginseng by *Lactobacillus bulgaricus* resulted in the transformation of saponins, including Rg1, Re, Rb1, Rc, Rb2, S-Rg2, and Rd, into several other saponins. By analyzing the retention times of ginsenoside reference compounds, the following ginsenosides were identified: R-Rg2, S-Rg3, R-Rg3, Rk1, Rg5, and CK.

### Analysis of effects of freeze-dried fermented ginseng powder on body weights of mice

**Fig. 2:**
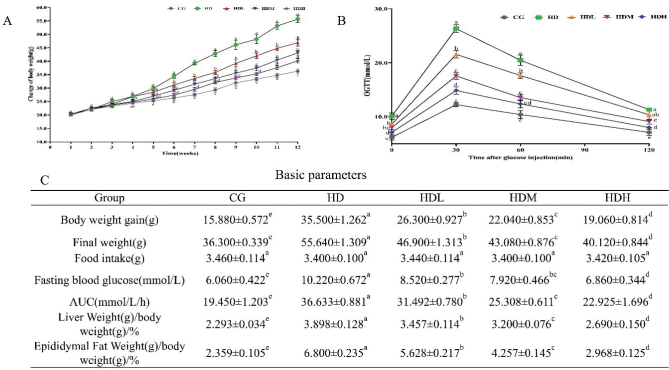
Effects of lyophilized fermented ginseng powder on body weight, fasting blood glucose levels, and body weights of experimental mice. (A) change of body weight, (B) oral glucose tolerance test (OGTT), and (C) basic parameters. Duncan’s Multiple Extreme Difference Test was employed, and differences between the superscript letters (a, b, c, d, and e) in the graphs were statistically significant (*P* < 0.05).

The effects of lyophilized fermented ginseng powder on the body weight of mice are shown in Fig. 2A. After 4 weeks of feeding with a HFD, the body weight of the mice gradually increased, with statistically significant differences observed between the HD group and the other groups. After 12 weeks of continuous gavage treatment with lyophilized fermented ginseng powder, mice in the HD group exhibited the most rapid increase in body weight compared to those in the other groups (Fig. 2C). Fasting blood glucose and body weight in the HDL, HDM, and HDH groups were significantly reduced compared to the HD group (Fig. 2C). Epididymal fat and liver weights were significantly lower in the HDL, HDM, and HDH groups compared to the HD group (Fig. 2C). No significant differences in food intake were observed (Fig. 2C). Both the OGTT and the area under the curve (AUC) demonstrated improved glucose tolerance in the HDL, HDM, and HDH groups compared to the HD group (Fig. 2B, C).

### Effect of fermented ginseng lyophilized powder on serum lipid and inflammation-related indices in mice

The effects of lyophilized fermented ginseng powder on serum biomarkers in mice are shown in Table 1. Compared with the HD group, the serum levels of TC were significantly lower in the CG, HDL, HDM, and HDH groups. In contrast, the levels of TG and LDL-C were lower with the change in dosage, and there was no significant difference between the HDH and CG groups. On the contrary, HDL-C content was significantly increased. Serum levels of IL-1, IL-6, and TNF-α were significantly lower in the CG, HDL, HDM, and HDH groups compared to the HD group.

**Table 1:** Effects of lyophilized fermented ginseng powder on serum lipid and inflammation-related indices in mice

Group	CG	HD	HDL	HDM	HDH
TC (mmol/L)	2.334 ± 0.701^e^	4.174 ± 0.246^a^	3.545 ± 0.107^b^	3.092 ± 0.091^c^	2.639 ± 0.171^d^
TG (mmol/L)	0.433 ± 0.358^de^	1.151 ± 0.088^a^	0.911 ± 0.077^b^	0.808 ± 0.050^c^	0.502 ± 0.075^d^
LDL-C (mmol/L)	1.225 ± 0.373^de^	2.913 ± 0.077^a^	2.181 ± 0.097^b^	1.796 ± 0.049^c^	1.299 ± 0.040^d^
HDL-C (mmol/L)	2.384 ± 0.068^a^	1.321 ± 0.0.021^e^	1.651 ± 0.072^d^	1.929 ± 0.029^c^	2.157 ± 0.076^b^
IL-1 (pg/mL)	141.077 ± 3.770^de^	305.756 ± 10.362^a^	230.377 ± 9.200^b^	170.735 ± 11.701^c^	146.509 ± 6.346^d^
TNF-α (pg/mL)	110.321 ± 6.070^e^	304.219 ± 10.282^a^	227.178 ± 5.850^b^	156.809 ± 11.440^c^	126.879 ± 12.080^d^
IL-6 (pg/mL)	19.162 ± 1.241^e^	35.831 ± 3.116^a^	30.025 ± 1.763^b^	25.319 ± 1.357^c^	23.145 ± 1.827^cd^

Duncan’s Multiple Extreme Difference Test was used, and differences between the superscript letters (a, b, c, d, and e) in the graphs were statistically significant (*P* < 0.05).

### Effects of lyophilized fermented ginseng powder on fatty tissue of liver and epididymis in mice

**Fig. 3:**
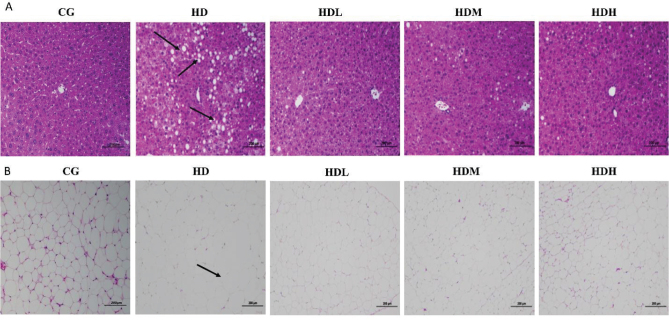
Histopathological analysis of murine epididymal adipose and liver tissue. (A) Liver section from obese mice exhibiting signs of steatosis, with numerous lipid droplets accumulating in the hepatocytes (arrows). The tissue was stained with H&E (20 ×); (B) Epididymal adipose tissue section showing hypertrophic adipocytes and increased lipid droplet size in obese mice (arrows). The tissue was stained with H&E (20 ×).

Murine liver tissues were stained by H&E and observed in paraffin sections as shown in Fig. 3A. Compared with the HD group, mice in the CG, HDL, HDM, and HDH groups showed a significant reduction in intracellular hepatocellular steatosis, obvious decrease in the number of intracellular lipid droplets, and a reduction in the degree of steatosis after the intervention of gavage with the lyophilized ginseng powder from the fermented ginseng group; the higher the dosage, the more pronounced the effect was.

**Fig. 4:**
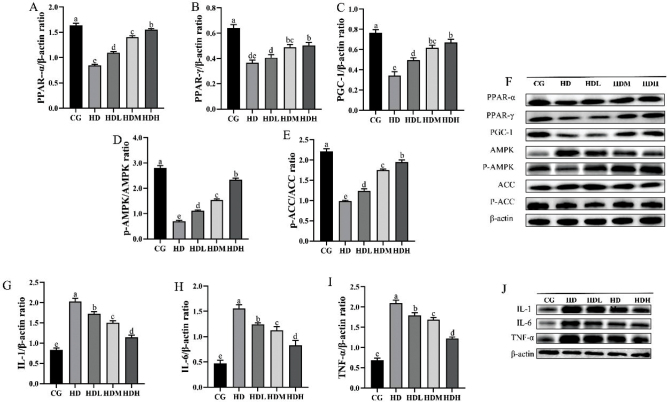
Effects of lyophilized fermented ginseng powder on lipid metabolism and inflammation-related proteins in mouse liver tissue. (A–E) PPAR-α, PPAR-γ, PGC-1, P-AMPK/AMPK, and P-ACC/ACC protein expression gray value. (F) PPAR-α, PPAR-γ, PGC-1, P-AMPK, AMPK, P-ACC, and ACC protein expressions in liver tissue. (G–I) IL-1, IL-6, and TNF-α protein expression gray value. (J) IL-1, IL-6, and TNF-α protein expressions in liver tissue. Differences between the superscript letters (a, b, c, d, and e) in the graphs were statistically significant (*P* < 0.05) using Duncan’s Multiple Extreme Difference test.

The adipose tissue of mouse epididymis was stained with H&E and microscopically observed after paraffin sectioning, as shown in Fig. 3B. After the intervention of lyophilized fermented ginseng powder, the adipocytes of mice in the CG, HDL, HDM, and HDH groups were much smaller and more uniform in size than those in the HD group, and the effect of the high dose was greater than that of the medium dose.

### Effects of lyophilized fermented ginseng powder on lipid metabolism and expression of inflammation-related proteins in liver of mice

**Fig. 5:**
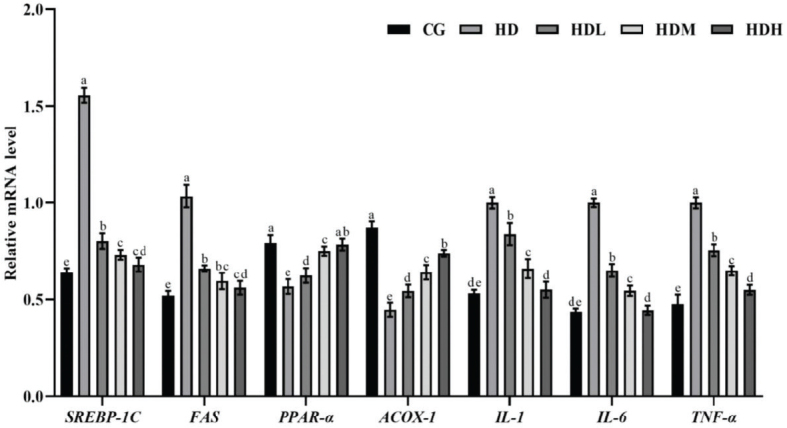
Effects of lyophilized fermented ginseng powder on genes related to lipid metabolism in murine liver tissue. Relative mRNA levels of genes related to lipid formation and lipid oxidation in liver tissues were tested by Duncan’s Multiple Extreme Difference Test, and the differences between the superscript letters in the graphs (a, b, c, d, and e) were statistically significant (*P* < 0.05).

After gavage intervention in mice using freeze-dried fermented ginseng powder, the expression levels of lipid metabolism and inflammation-related proteins in mouse liver tissues were shown in Fig. 5. Compared with the HD group, freeze-dried fermented ginseng powder significantly increased the expression of each of the related proteins, PPAR-α, PPAR-γ, PGC-1, P-AMPK/AMPK, and P-ACC/ACC (Fig. 5–E). Compared to the HD, and CG, the expression levels of IL-1, IL-6, and TNF-α proteins in the HDL, HDM, and HDH and CG groups were significantly decreased (Fig. 5G–I), and this effect became increasingly obvious with higher doses.

### Effects of lyophilized fermented ginseng powder on lipogenic and inflammatory gene ex-pression in mice

Gene expression results showed (Fig. 5) that *SREBP-1c* and *FAS* mRNA expression levels were reduced in the CG, HDL, HDM, and HDH groups compared to the HD group, suggesting that fatty acid synthesis was reduced in these animals. *PPAR-α* mRNA expression was significantly increased in the CG, HDL, HDM, and HDH groups compared with the HD group. Elevated *ACOX-1* mRNA expression pro-moted fatty acid metabolism by regulating fatty acid metabolism processes, which resulted in an increase in intracellular fatty acid storage in mice. The relative expression levels of inflammation-related genes (*TNF-α, IL-1, IL-6*) were significantly lower in the CG, HDL, HDM, and HDH groups compared with the HD group, and varied with dose, suggesting that the intervention of lyophilized fermented ginseng powder could inhibit the production of inflammatory factors.

## Discussion

The present study investigates the hypoglycemic and lipid-lowering effects of lyophilized fermented ginseng powder in mice fed an HFD. Our findings indicate that fermented ginseng powder, enriched with rare ginsenosides, significantly improves glucose tolerance and modulates serum lipid profiles, thereby exerting a potential therapeutic effect against diet-induced obesity and associated metabolic disorders. The OGTT results demonstrated that the administration of fermented ginseng powder markedly improved glucose tolerance in the HDL, HDM, and HDH groups. This observation is consistent with previous studies suggesting that ginsenosides, particularly those derived from fermented ginseng, can enhance insulin sensitivity and glucose uptake by skeletal muscles and adipocytes (18, 19). The improvement in glucose tolerance may be attributed to the activation of AMPK signaling, which plays a crucial role in regulating glucose and lipid metabolism. Previous studies have shown that activation of AMPK enhances glucose uptake and utilization, thereby improving insulin sensitivity and reducing hyperglycemia (20). In addition to the hypoglycemic effects, fermented ginseng powder also positively affected serum lipid profiles. Specifically, the administration of lyophilized ginseng powder led to significant reductions in TC levels and a decrease in TG and LDL-C levels in the HDL, HDM, and HDH groups, compared to the HFD group. These findings agree with previous studies, which have demonstrated that ginsenosides can regulate lipid metabolism and reduce the risk of atherosclerosis by modulating lipoprotein cholesterol levels (21, 22). The observed reduction in serum TC, TG, and LDL-C levels suggests that fermented ginseng powder may have a lipid-lowering effect, potentially through the modulation of genes involved in lipid syntheses and uptake, such as SREBP-1c and FAS (23). Moreover, our results showed that HDL-C levels were significantly increased in the fermented ginseng treatment groups. HDL-C is known as ‘good’ cholesterol due to its role in reverse cholesterol transport, where it facilitates the removal of excess cholesterol from peripheral tissues to the liver for excretion (24). The elevation of HDL-C in the treatment groups suggests that fermented ginseng powder may enhance this protective mechanism, potentially reducing the risk of cardiovascular diseases associated with obesity and metabolic syndrome. In addition to the effects on glucose and lipid metabolism, fermented ginseng powder also significantly reduced serum levels of pro-inflammatory cytokines, including IL-1, IL-6, and TNF-α. Chronic inflammation is a well-established factor in the pathogenesis of obesity, insulin resistance, and metabolic disorders (25). Our findings are consistent with previous studies demonstrating that ginsenosides possess anti-inflammatory properties, likely by inhibiting inflammatory pathways (26). The reduction in inflammatory markers in the treatment groups further supports the potential of fermented ginseng powder in mitigating obesity-induced inflammation.

In this study, we evaluated the effect of fermented ginseng powder on HFD-induced obesity in mice, focusing on its impact on liver steatosis and adipose tissue morphology. The results demonstrated that fermented ginseng powder significantly ameliorated the adverse effects of a HFD, suggesting its potential in managing obesity and related metabolic disorders. Firstly, H&E staining of murine liver tissues revealed a marked reduction in hepatocellular steatosis following the administration of fermented ginseng powder. This finding aligns with previous studies that suggest ginseng and its fermented derivatives can protect the liver from lipid accumulation and mitigate non-alcoholic fatty liver disease (27, 28). In addition, the histological examination of epididymal adipose tissue further supported the anti-obesity effect of fermented ginseng powder. H&E staining revealed that the adipocytes in the CG, HDL, HDM, and HDH groups were significantly smaller and more uniform in size compared to those in the HD group. The high-dose intervention produced the most notable changes in adipocyte morphology, demonstrating the dose-dependent effect of fermented ginseng powder. This observation is consistent with the known ability of ginseng to modulate adipogenesis and promote the reduction of fat cell size and number in the context of obesity (29, 30).

In this study, we investigated the effects of freeze-dried fermented ginseng powder on lipid metabolism and inflammation-related proteins in liver tissues of mice induced by a HFD. The results demonstrated that fermented ginseng powder significantly modulated the expression of key proteins involved in lipid metabolism and inflammation, providing insights into its potential therapeutic mechanisms for obesity-related metabolic disorders. Firstly, we observed that fermented ginseng powder increased the expression of several proteins related to lipid metabolism, including PPAR-α, PPAR-γ, PGC-1, P-AMPK/AMPK, and P-ACC/ACC. These proteins regulate fatty acid oxidation, lipogenesis, and overall energy metabolism. Specifically, PPAR-α and PPAR-γ are important transcription factors involved in regulating lipid homeostasis, while PGC-1 is a coactivator that promotes mitochondrial biogenesis and oxidative metabolism (31). AMPK is a key energy-sensing enzyme that, when activated by P-AMPK, enhances fatty acid oxidation and suppresses lipogenesis (32). ACC is involved in the regulation of fatty acid synthesis, and its P-ACC inhibits fatty acid biosynthesis. The increased expression of these proteins in the liver tissues of mice treated with freeze-dried fermented ginseng powder suggests that the intervention promotes lipid catabolism and inhibits lipid accumulation, which may contribute to its anti-obesity effects (33). In addition to lipid metabolism regulation, the expression levels of pro-inflammatory cytokines such as IL-1, IL-6, and TNF-α were significantly decreased in the HDL, HDM, and HDH groups compared to the HD and CG groups. These cytokines are known to play a central role in the inflammatory processes associated with obesity and metabolic disorders. Chronic low-grade inflammation is a hallmark of obesity, and the reduction of these inflammatory markers after fermented ginseng powder intervention suggests that it may exert an anti-inflammatory effect. Moreover, this effect was more pronounced at higher doses of fermented ginseng powder, indicating a dose-dependent response (34).

In this study, gene expression analysis provided valuable insights into the molecular mechanisms underlying the effects of lyophilized fermented ginseng powder on HFD-induced obesity in mice. The results revealed significant changes in the expression of genes related to lipid metabolism and inflammation, further supporting the beneficial effects of fermented ginseng powder in modulating adipogenesis and reducing inflammation. First, the expression of genes involved in fatty acid synthesis, such as *SREBP-1c* and *FAS*, was significantly reduced in the CG, HDL, HDM, and HDH groups compared to the HD group. *SREBP-1c* is a key regulator of lipogenesis, and FAS is an enzyme involved in the synthesis of fatty acids. The downregulation of these genes suggests that fermented ginseng powder reduces fatty acid synthesis in liver tissues, which may contribute to a decrease in hepatic lipid accumulation. These findings are consistent with previous studies that demonstrated the ability of ginseng and its derivatives to modulate lipid synthesis and promote lipid homeostasis (27, 35). Previous studies have also shown that monascus fermented ginseng may regulate lipid metabolism through PPAR and SREBP signaling pathways (36). In contrast, the mRNA expression of *PPAR-α*, a transcription factor that regulates fatty acid oxidation, was significantly increased in the CG, HDL, HDM, and HDH groups compared to the HD group. *PPAR-α* activation promotes the expression of genes involved in fatty acid oxidation, such as *ACOX-1*, which is essential for the catabolism of long-chain fatty acids. The elevated expression of *ACOX-1* in the fermented ginseng powder-treated groups suggests enhanced fatty acid metabolism, which may facilitate the reduction of fat accumulation in the liver and adipose tissue (37, 38). These results support the hypothesis that fermented ginseng powder promotes lipid metabolism by stimulating fatty acid oxidation and reducing lipogenesis, thus contributing to the alleviation of obesity. Additionally, the gene expression analysis showed a significant reduction in the mRNA levels of pro-inflammatory cytokines, including *TNF-α*, *IL-1*, and *IL-6*, in the CG, HDL, HDM, and HDH groups compared to the HD group. These inflammatory markers are known to be overregulated in obesity and contribute to chronic low-grade inflammation, which exacerbates metabolic disorders. The observed decrease in the expression of these inflammatory genes suggests that fermented ginseng powder has anti-inflammatory effects, potentially by reducing the inflammatory response associated with obesity. Furthermore, the reduction in inflammation was dose-dependent, with higher doses of fermented ginseng powder showing more pronounced effects. This finding is in agreement with previous studies that highlighted the anti-inflammatory properties of ginseng and its active components, which may play a key role in mitigating obesity-related inflammation (39, 40).

## Conclusions

This study provides novel insights into the therapeutic potential of lyophilized fermented ginseng powder enriched with rare ginsenosides in the context of HFD-induced obesity. Our findings demonstrate that fermented ginseng powder significantly modulates lipid metabolism and inflammatory pathways in obese mice, highlighting its ability to reduce lipid accumulation and adipocyte size while improving the lipid profile. Notably, the intervention enhanced the expression of key regulators of adipogenesis and metabolism, including PPAR-α, PPAR-γ, and AMPK, while attenuating the expression of pro-inflammatory cytokines. These results not only support the role of fermented ginseng powder in mitigating obesity-induced metabolic dysregulation but also open avenues for its potential as a therapeutic agent in managing obesity and associated metabolic diseases. Future studies should explore the mechanistic pathways underlying its effects and evaluate its safety and efficacy in human clinical trials.

## Data Availability

The data presented in this study are available on request from the corresponding author due to privacy and ethical concerns.
